# Non-Gated Laser Induced Breakdown Spectroscopy Provides a Powerful Segmentation Tool on Concomitant Treatment of Characteristic and Continuum Emission

**DOI:** 10.1371/journal.pone.0103546

**Published:** 2014-08-01

**Authors:** Ashwin Kumar Myakalwar, Narahara Chari Dingari, Ramachandra Rao Dasari, Ishan Barman, Manoj Kumar Gundawar

**Affiliations:** 1 Advanced Centre of Research in High Energy Materials (ACRHEM), University of Hyderabad, Gachibowli, Hyderabad, Andhra Pradesh, India; 2 Laser Biomedical Research Center, G. R. Harrison Spectroscopy Laboratory, Massachusetts Institute of Technology, Cambridge, Massachusetts, United States of America; 3 Department of Mechanical Engineering, Johns Hopkins University, Baltimore, Maryland, United States of America; Washington State University, United States of America

## Abstract

We demonstrate the application of non-gated laser induced breakdown spectroscopy (LIBS) for characterization and classification of organic materials with similar chemical composition. While use of such a system introduces substantive continuum background in the spectral dataset, we show that appropriate treatment of the continuum and characteristic emission results in accurate discrimination of pharmaceutical formulations of similar stoichiometry. Specifically, our results suggest that near-perfect classification can be obtained by employing suitable multivariate analysis on the acquired spectra, without prior removal of the continuum background. Indeed, we conjecture that pre-processing in the form of background removal may introduce spurious features in the signal. Our findings in this report significantly advance the prior results in time-integrated LIBS application and suggest the possibility of a portable, non-gated LIBS system as a process analytical tool, given its simple instrumentation needs, real-time capability and lack of sample preparation requirements.

## Introduction

One of the major objectives of Process Analytical Technologies, conceptualized by the U.S. Food and Drug Administration in the last decade, is the development of novel sensor devices that can be incorporated in the manufacturing process loop to enable in-process material characterization [1]. Such a method can assist in better monitoring each step of the formulation development and manufacturing process and, therefore, in real-time control of the process itself. However, there is a lack of analytical tools that can perform rapid on-line determination of the consistency of the drug constituents (especially the active pharmaceutical ingredient, API) in order to ensure the potency, purity and bioavailability of the final product. Laser-induced breakdown spectroscopy (LIBS) is an emerging instrument in the analytical toolkit, as it can provide real-time analysis with minimal or no sample preparation [2]–[5]. In LIBS, the emission from the plasma plume, induced by laser photons, forms the basis for extraction of analytical information about the material under investigation [6]. The deionization radiation is primarily characteristic of the elemental composition, although emission from molecular fragments may also be observed [7]. Because of its real-time diagnostic capability, LIBS can be potentially used for testing a larger number of samples in comparison to existing analytical tools (*e.g.* high performance liquid chromatography (HPLC)), with the additional possibility of high-resolution surface mapping and depth profiling. However, despite these intrinsic advantages, LIBS systems have hitherto not been employed for online process monitoring [4], [6]. The primary bottlenecks towards more extensive usage, particularly for screening applications, is the lack of robustness and resource-intensive, unwieldy nature of the conventional LIBS spectrometers that use gated intensified detectors (primarily intensified charge-transfer devices (ICCD)) for spectral recording. This is further compounded by the large spatial footprint and weight of these systems as well as the considerable maintenance and technical expertise required for its routine use.

Application of gated detection is based on the prevailing view that discrimination against the early “uncharacteristic” continuum signal (from radiative recombination and Bremsstrahlung emission) is critical for quantitative analysis [8]. As a consequence, most of the reports have focused on a suitable time window of acquisition where the condition of local thermodynamic equilibrium (LTE) is satisfied [9]. While this perspective is largely justified for trace element analysis as well as suppression of matrix effects in certain specimen [10], such gating may not be necessary for classification applications even when dealing with samples of similar chemical composition. We have suspected that it is possible to obtain similar levels of performance without gated detection by appropriately utilizing the features across the entire spectral window of collection. In fact, the distinctiveness of the plasma produced by different samples may result in subtle differences in the broad continuum signals, which in turn could positively aid performance of the classification models. Classical ratiometric analysis based on a study of a few channel (wavelength) traces has limited capability of dealing with segmenting such spectral datasets due to the overlap of the continuum and characteristic emission signals and to the impossibility of detecting interfering species in the measured signal. Our prior experience with analysis of Raman spectra acquired from biological samples in the presence of substantive luminescence backgrounds [11] further strengthens our belief that multivariate classification of non-gated LIBS spectra can be successfully implemented despite the presence of the continuum background signals. Here, we seek to investigate the capability of non-gated LIBS for differentiation of pharmaceutical samples of similar elemental composition by concomitant treatment of continuum and characteristic emission. This work extends the recent efforts in understanding the role of continuum radiation in LIBS [12] and its application in metal alloy identification [13] to analysis of organics (*e.g.* API) of similar composition.

In this article, we report the application of LIBS measurements to classify pharmaceutical formulations in solid dosage forms, without employing gated detectors or echelle spectrographs. Our non-gated LIBS measurements reveal that despite the presence of substantive continuum emission, the acquired signals exhibit subtle, but consistent, changes in spectral features. By correlating the spectra with the corresponding class of samples, we have developed predictive models based on soft independent modeling of class analogy (SIMCA), artificial neural networks (ANN) and partial least squares discriminant analysis (PLSDA). Our multivariate analysis shows very high diagnostic power with correct classification accuracy levels in excess of 90% with SIMCA and 100% with ANN and PLSDA, even when measurements are performed under ambient air conditions. Taken together with our recent development of a sensitive and robust support vector machine platform [14], these results provide a powerful toolkit in the minimally perturbative process monitoring (on-line and at-line) and quality control domains in the pharmaceutical industry. While this report provides a proof of concept demonstration of the latent diagnostic power of a simple portable LIBS system, we are currently in the process of validating our results on a larger matrix of samples including specimen spiked with trace foreign elements to mimic the scenario of counterfeit drugs. Ultimately, we envision that the substantive advantages of using non-gated detection in LIBS in terms of cost, maintenance and simpler instrumentation will enable its ready translation to compact devices tailored for the pharmaceutical and food industries as well as for forensic and biological specimen analysis.

## Materials and Methods

The details of the experimental methods, instrumentation and data analysis steps are provided in File S1. Briefly, a set of pharmaceutical samples (namely Cetirizine dihydrochloride, Metformin hydrochloride, Cipro pure and Ciprofloxacin hydrochloride) was acquired from the local drug manufacturer in powder form. To simulate the in-line testing of tablets, the fine powder material was pressed into *ca.* 1 cm diameter pellets by a die-hydraulic press combination. The LIBS system used a frequency-doubled Nd:YAG laser (λ_ex_ = 532 nm, 7 ns pulse width, Spit light 1200, InnoLas LaserGmbH, Germany) for excitation and the emission signal was recorded using a non-gated spectrometer (Ocean Optics, MAYA 2000). Notably, the detector unit comprised of a conventional Czerny-Turner spectrograph for dispersion instead of the higher resolution echelle spectrograph allowing us to assess a lower bound for the classification performance. The samples were subjected to *ca.* 6 mJ of laser energy. All of the LIBS spectra were collected under ambient conditions. The data can be found in Data S1. A motorized XY stage was employed to enable a fresh portion of the sample to be interrogated after acquiring multiple spectra from a single location.

Subsequent to acquisition and pre-processing of the spectral data, principal component analysis (PCA) was employed as a data exploration and dimensionality reduction step. For quantifying the classification ability of the LIBS dataset, we selected SIMCA, PLS-DA and ANN as representative methods. Chemometric calculations, including dendrogram analysis-based outlier detection and subsequent classification, were conducted using MatlabR2010b (Math Works, Natick, MA). Following removal of outliers, a total of 403 LIBS spectra were used in the classification analysis, with more than 90 spectra from each of the four pharmaceutical formulations.

## Results

**Figure 1:**
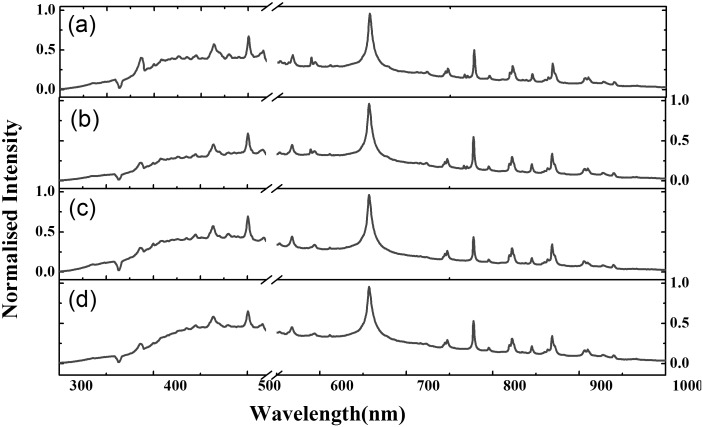
Representative LIBS spectra acquired from the pharmaceutical formulation investigated in this report. (a) Cetirizine dihydrochloride; (b) Cipro pure; (c) Metformin hydrochloride; (d) Ciprofloxacin hydrochloride. Intensity on the y-axis is normalized with respect to the characteristic hydrogen emission peak at 656 nm.

Figure 1 shows representative spectra acquired from the Cetirizine dihydrochloride, Cipro pure, Metformin hydrochloride, and Ciprofloxacin hydrochloride pellets. The discontinuity in the wavelength axis is due to the omission of a 30 nm wide band surrounding the 532 nm laser line, which otherwise causes significant interference in the non-gated signal. The corresponding intensity values are disregarded from the ensuing analysis. From Fig. 1, it is notable how similar the LIBS spectra from each of the pharmaceutical samples are, due to the relatively similar formulation of the API. Further, any differences that may exist (for example, the absence of oxygen in metformin hydrochloride) are obviated by the presence of the corresponding element(s) in air. A complete listing of the prominent peaks in Fig. 1 is provided in Table S1. Evidently, the early continuum emission (present as broad featureless background) constitutes a substantive component of the acquired spectra. Despite the apparent featureless nature, however, it is possible that the subtle differences in the continuum emission signals may provide discriminatory power – particularly when viewed in light of the similarity of the characteristic emission lines in Fig. 1.

**Figure 2:**
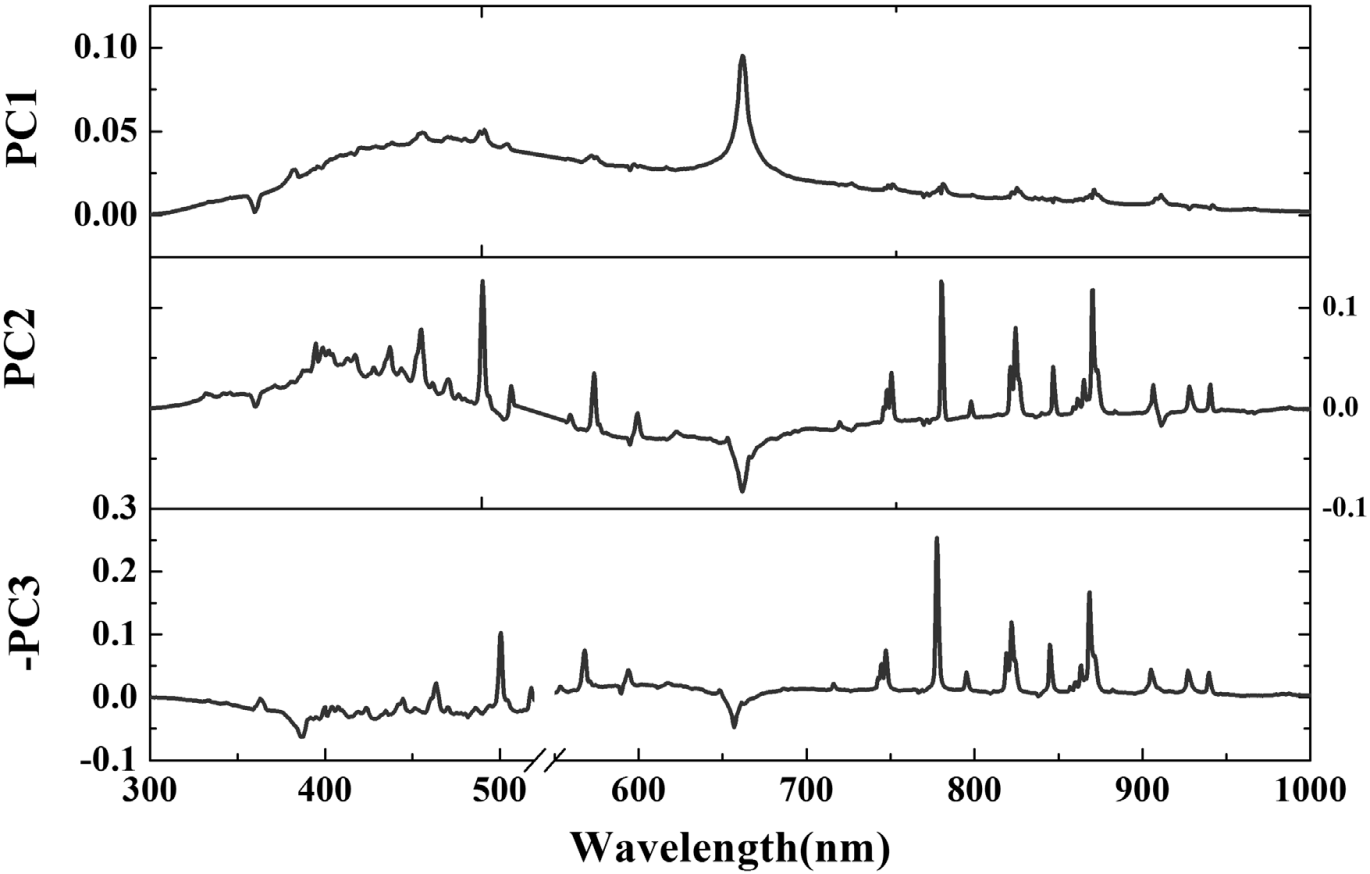
Plot of the first three principal components corresponding to the entire spectral dataset acquired for all classes. These three principal components, combined, explain 96.5% of the net variance in the dataset.

To systematically investigate the presence of subtle differences between the LIBS spectra of each type of pharmaceutical sample, principal component analysis (PCA) was used. The principal components are linear combinations of the acquired signals and capture the spectral variance in a reduced dimensional space. Figure 2 shows the first three principal components, which together account for 96.5% of the net variance in the spectral dataset. In particular, the first two PCs explain 93% of the variance present in the dataset, with the first one contributing 84.3%. Expectedly, the subsequent PCs after the first three are mostly dominated by noise. We observe that PC1 shows only a single dominant hydrogen emission line at *ca.* 657 nm on a broad background that can be ascribed to the early continuum emission. PC2 and PC3 exhibit mostly characteristic emission lines including those at *ca.* 479.45 nm (chlorine), 500 nm (nitrogen), 567 nm (nitrogen), 747 nm (nitrogen), 777 nm (oxygen) and 868 nm (oxygen). The primary differences between PC2 and PC3 are in the relative intensities of these lines and also in the presence of a small background for PC2, especially in the lower wavelength region.

**Figure 3:**
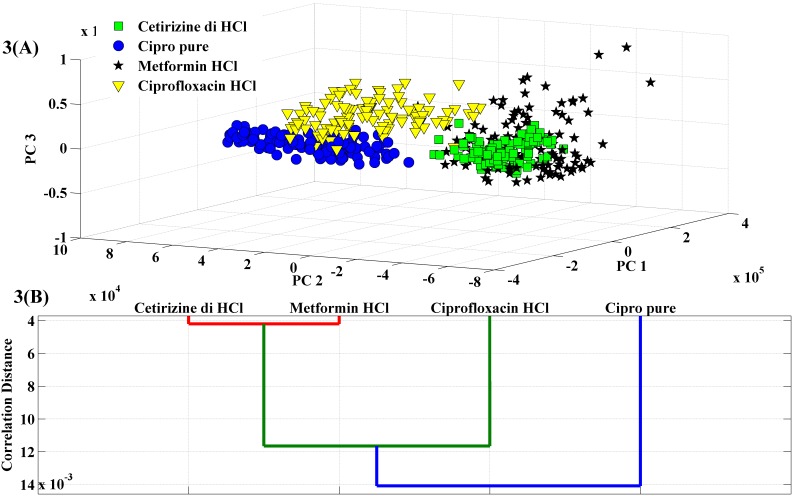
Discrimination of pharmaceutical samples based on their LIBS spectra. (**A**) Scores plot corresponding to principal components 1, 2 and 3 for the spectral dataset acquired from the four samples. The data points corresponding to Cetirizine dihydrochloride, Cipro pure, Metformin hydrochloride and Ciprofloxacin hydrochloride are indicated by green squares, blue circles, black asterisks and yellow inverted triangles, respectively. (**B**) Hierarchical clustering using the dendrogram representation for LIBS spectra acquired from the 4 sets of pharmaceutical samples.

The corresponding scores plot for PC 1, PC 2 and PC 3 is provided in Fig. 3(A). From the figure, it is evident that the samples of each class tend to form a cluster and in general appear to show good separation from the other classes. In particular, Cetirizine dihydrochloride, Cipro pure and Ciprofloxacin hydrochloride are clearly distinguishable based on the PC scores obtained from the acquired LIBS spectra. However, the scatter in the PC scores for the Metformin hydrochloride spectra makes its separation more challenging, particularly from the Cetirizine dihydrochloride cluster. The scatter in the PC scores arises primarily from the inhomogenity of the fabricated pellets as well as the potential non-sample specific variance introduced by non-gated detection. Additionally, the overlap in the the Metformin hydrochloride and the Cetirizine dihydrochloride clusters can also be attributed to the absence of any distinguishing element between the two formulations, *i.e.* both formulations in ambient air conditions exhibit emission lines of carbon, hydrogen, oxygen, nitrogen and chlorine. This is in contrast to the Cipro pure and Ciprofloxacin hydrochloride samples that contain fluorine in their corresponding API. Nevertheless, in totality, this exploratory data analysis of the non-gated LIBS data reveals that the chemical basis in the form of PCs gives rise to a substantive degree of separation of the data points corresponding to a particular pharamceutical formulation – which is promising for the development of models for classifying and screening these and similar pharmaceutical tablets.

To ensure suitable quality of the data for the development of classification algorithms, hierarchical clustering by means of dendrogram analysis was first pursued. In this case, we exploit the grouping of the objects in a dendrogram to detect outliers based on the presence of isolated branches. Moreover, dendrogram analysis was also used to obtain class similarity between the different pharmaceutical formulations. In this case, the mean spectrum for each formulation was computed from all the spectra acquired from that formulation barring the detected outlier spectra. Figure 3(B) shows the resultant dendrogram obtained by analyzing the mean spectrum of the four formulations. This corroborates the PCA findings as one clearly observe that the Metformin hydrochloride exhibits spectral similarity to Cetirizine dihydrochloride in comparison to the other formulations.

In order to evaluate the suitability of the non-gated LIBS data for classification purposes, SIMCA was chosen to develop the discrimination algorithm. Specific to the SIMCA model development, 70% of the samples were designated as training data while the rest of the samples (30%) were held out of the model building and served as the test set. It was ensured that the spectra corresponding to each pharmaceutical formulation were split as per these representative percentage values. In order to obtain a representative estimate of the rates of correct classification, misclassification and unclassification, 100 independent iterations were performed by re-splitting the entire data into training and test sets. Here, the model is judged to be sensitive if the correct classification rate is high and the unclassification rate is low.

Table 1 gives the results of the classification analysis for the SIMCA-derived discrimination model for the four pharmaceutical formulations, based on the 3σ unclassification threshold (Supporting information). The misclassification rate is observed to be 0%, irrespective of the pharamceutical formulation in question. The correct classification accuracy varies from 82% (Metformin hydrochloride) to 100% (Cetirizine dihydrochloride). Based on the PCA and dendrogram analysis, the relatively lower classification accuracy (and correspondingly, the higher unclassification rate) of the Metformin hydrochloride sample is not surprising. The inherent scatter of the sample data seems to be the predominant factor in the relatively inferior performance of the SIMCA model for Metformin hydrochloride and Cipro pure classification with unclassification rates observed to be 18% (Metformin hydrochloride) and 14% (Cipro pure), respectively. On average, the SIMCA-derived model provides a correct classification rate of 91%, which suggests that the non-gated LIBS spectral measurements, even under ambient air conditions, provides sufficient information for sensitive discrimination of the studied formulations. It is worth mentioning that these results are comparable to our previous results obtained from gated LIBS spectra of similar pharmaceutical samples [14], [15] - indicating that the presence of the continuum emission signals does not hinder the statistical performance, especially on application of suitable multivariate algorithms. To the best of our knowledge, this provides the first experimental demonstration of the suitability of non-gated data for classification of complex samples with the principal constituents (i.e. the APIs) having similar composition.

**Table 1:** SIMCA classification results obtained from the test samples over 100 iterations.

Average rate of…	Correct classification	Misclassification	Unclassification
Cetirizine dihydrochloride	1.00	0.00	0.00
Cipro pure	0.86	0.00	0.14
Metformin hydrochloride	0.82	0.00	0.18
Ciprofloxacin hydrochloride	0.96	0.00	0.04
**Average**	0.91	0.00	0.09

Finally, to more comprehensively examine the classification capability of the acquired non-gated data, PLS-DA and ANN models were also constructed. For PLS-DA, a single “global” model is obtained for classification, in contrast to the independent PCA submodels developed for SIMCA analysis. Using this single global PLS-DA model, we were able to obtain 100% correct classification accuracy for all formulations (Table 2). While the underlying principle of PLS-DA to obtain maximum separation between classes (as opposed to modeling the maximum variance in each individual class in SIMCA) may be partially responsible for the improvement, the presence of dataset-specific factors cannot be neglected. For example, we had previously observed for the corresponding gated data that SIMCA was marginally more sensitive, even though PLS-DA was significantly more robust in classifying unknown samples [14]. Furthermore, application of ANN also yields no misclassification or unclassification (Table 3). Given the nonlinear nature of the ANN algorithm and the potential interferent sources in the non-gated data, the enhanced performance in this case, with respect to SIMCA, is not wholly unexpected (e.g. application of support vector machine (SVM) derived segmentation model greatly improved the sensitvity and robustness metrics in our gated data [14]. Variability in the continuum emission, plasma self-absorption and matrix effects could contribute towards such nonlinear changes in intensity ratios in the features of interest.

**Table 2:** ANN classification results obtained from the test samples over 100 iterations.

Average rate of…	Correct classification	Misclassification	Unclassification
Cetirizine dihydrochloride	1.00	0.00	0.00
Cipro pure	1.00	0.00	0.00
Metformin hydrochloride	1.00	0.00	0.00
Ciprofloxacin hydrochloride	1.00	0.00	0.00
**Average**	1.00	0.00	0.00

**Table 3:** PLSDA classification results obtained from the test samples over 100 iterations.

Average rate of…	Correct classification	Misclassification	Unclassification
Cetirizine dihydrochloride	1.00	0.00	0.00
Cipro pure	1.00	0.00	0.00
Metformin hydrochloride	1.00	0.00	0.00
Ciprofloxacin hydrochloride	1.00	0.00	0.00
**Average**	1.00	0.00	0.00

## Discussion

In addition to quantifying the classifier performance based on non-gated LIBS spectra, it is imperative to precisely understand the impact of the continuum emission background. As alluded to above, the prevailing thought in the LIBS community has centered around the uncharacteristic nature of the continuum emission, as it emanates from radiative recombination and Bremsstrahlung emission that do not depend on the identity of the element/ion [16]. Considerable contemporary attention has been focused on the choice of a proper delay time when the ratio of line emission to background (continuum) emission is very high, since “only the lines emission from the plume is important for the compositional analysis of the sample target” [6].

To test this hypothesis, we have compared the aforementioned results using non-gated LIBS spectra to those where the continuum background is removed using a lower order polynomial. Since the continuum background is broad and featureless, previous investigators have employed several numerical post-processing schemes to approximate and remove it from the acquired spectral data [17], [18]. Here, the background of the LIBS spectra was removed by application of an iterative least squares-based curve-fitting algorithm that uses a polynomial (6^th^ order for our dataset) with non-negativity constraints [19]. This algorithm (and its variants) have been extensively used for addressing broad backgrounds in similar spectroscopic data featuring small characteristic peaks and large backgrounds [20], [21]. Representative background-removed LIBS spectra from each pharmaceutical formulation are shown in Fig. S1. Subsequent to background removal, all the spectra were subjected to the same protocol as described above, namely dendrogram analysis for outlier removal and SIMCA for development of the discrimination algorithm. Using 100 iterations on the partitioned test sets (30%), we observe that, in this case, the SIMCA-derived models provide an average correct classification rate of 88% with a corresponding misclassification and unclassification rates of 0% and 12%, respectively. Based on the average correct classification rates and the corresponding standard deviations for the non-gated LIBS data with and without background removal, the two-tailed *p*-value is computed to be less than 0.0001. By conventional criteria (*i.e.* rejection of null hypothesis at p-value less than 0.01), this difference can be considered to be extremely statistically significant. It is also observed that the reduction in correct classification rate relative to Table 1 is consistent for all the pharmaceutical formulations. The difference in correct classification rate on application of SIMCA on the two datasets could be attributed to: (A) the presence of diagnostic information in the continuum background; and/or (B) introduction of artifacts due to the background removal procedure that results in deterioration of model performance. Further experiments with a range of time delays (currently underway in our laboratories) are necessary to elucidate whether the continuum background may indeed aid specific classification analyses.

Finally, it is worth analyzing the influence of the additional noise component incorporated in the non-gated LIBS data due to the continuum emission background. Overall, the relative root-mean-square noise in a spectrum has two contributions, namely the constant fixed-pattern noise characterizing the non-uniform response of the CCD pixels and the shot noise. Given the high signal levels of LIBS data, the latter has lesser significance and can always be reduced relative to the signal level by acquiring for longer time periods. The better signal-to-noise characteristics of the CCD detectors employed for non-gated detection in relation to the ICCD used for gated detection also alleviates this problem. Additionally, employing a suitable flat-field correction scheme can eliminate the more relevant fixed-pattern noise for high intensity LIBS data. Recently, researchers have detailed a promising fixed-pattern noise removal approach that focuses on subtraction of spectra acquired before and after shifting of spectrometer grating [22], which can be suitably employed in future non-gated LIBS studies to enhance classifier performance.

### Concluding Remarks

In summary, we have proposed and demonstrated the potential of non-gated LIBS for identification and classification of pharmaceutical formulations with similar elemental compositions. It is observed that the non-gated spectra shows high efficacy in discrimination with an average correct classification rate of 91%. Importantly, from a scientific standpoint, we observe that the presence of the continuum background in the non-gated LIBS dataset does not impede the classification performance. We envision that the significant advantages of this detection method in terms of cost, maintenance and system portability, coupled with its sensitivity, will ideally complement the existing analytical technologies for determination of surface and internal distribution of API and excipients [23], in addition to verification of the drug content in the finished dosage form. This will enable its ready translation to compact devices tailored for various industrial applications like food, pharmaceutical, biological and forensic. The sensitivity of this LIBS approach can be further enhanced by implementing hybrid classification schemes, which feature a range of (linear and non-linear) chemometric strategies [24]. With further refinements in the classification methodologies as well as development of a hand-held LIBS monitor, the approach presented here can be extended for sensitive identification in other critical applications including detection of high energy and hazardous materials [25].

## Supporting Information

Figure S1 **Background corrected LIBS spectra acquired from the pharmaceutical formulation investigated in this report.** (a) Cetirizine dihydrochloride; (b) Cipro pure; (c) Metformin hydrochloride; (d) Ciprofloxacin hydrochloride. Intensity on the y-axis is normalized with respect to the characteristic hydrogen emission peak at 656 nm. The continuum background is approximated and removed using a least squares-based polynomial curve-fitting algorithm.
(TIF)

Table S1 **Elemental assignments of the major emission lines observed in the LIBS spectra acquired from the pharmaceutical samples used in this study.**
(DOC)

Data S1 **Supplementary data.**
(XLS)

File S1 **Supplementary Materials and Methods.**
(DOC)
