# Supplementary material for: Non-Gated Laser Induced Breakdown Spectroscopy Provides a Powerful Segmentation Tool on Concomitant Treatment of Characteristic and Continuum Emission
Source: PLoS One. 2014 Aug 1;9(8):e103546. doi: 10.1371/journal.pone.0103546 (PMC4118875; doi:10.1371/journal.pone.0103546)
Supplement: Table S1 — Elemental assignments of the major emission lines observed in the LIBS spectra acquired from the pharmaceutical samples used in this study. (DOC) [file pone.0103546.s002.doc]

| Wavelength(nm) | Element |
| --- | --- |
| 479.45 | Cl II |
| 500.51 | N II |
| 567.67 | N II |
| 593.178 | N II |
| 656.96 | H I |
| 742.3 | N I |
| 744.59 | N I |
| 747.24 | N I |
| 777.68 | O I |
| 833.81 | Cl I |
| 838.18 | Cl I |
| 845.16 | O I |
| 868.67 | O I |
