# Supplementary material for: Non-Gated Laser Induced Breakdown Spectroscopy Provides a Powerful Segmentation Tool on Concomitant Treatment of Characteristic and Continuum Emission
Source: PLoS One. 2014 Aug 1;9(8):e103546. doi: 10.1371/journal.pone.0103546 (PMC4118875; doi:10.1371/journal.pone.0103546)
Supplement: File S1 — Supplementary Materials and Methods. (DOC) [file pone.0103546.s004.doc]

**Supporting Information**

### Non-gated laser induced breakdown spectroscopy provides a powerful segmentation tool on concomitant treatment of characteristic and continuum emission

### Ashwin Kumar Myakalwar^a,#^, Narahara Chari Dingari^b,#^, Ramachandra Rao Dasari^b^, Ishan Barman^c^, Manoj Kumar Gundawar ^a*^

*^a^Advanced Centre of Research in High Energy Materials (ACRHEM), University of Hyderabad, Prof C R Rao Road, , Gachibowli, Hyderabad, Andhra Pradesh, 500046, India*

*^b^Laser Biomedical Research Center, G. R. Harrison Spectroscopy Laboratory, Massachusetts Institute of Technology, Cambridge, Massachusetts 02139, USA*

*^c^Department of Mechanical Engineering, Johns Hopkins University, Baltimore MD 21218, USA*

#Authors have made equal contributions

**^*^**Address all correspondence to:

G. Manoj Kumar; e-mail: manojsp@uohyd.ernet.in

**Table of Content**

Materials and Methods

- Sample Preparation
- Instrumentation and Data Acquisition
- Multivariate Data Analysis

Tables

Figures

References

**Materials and Methods**

*Sample Preparation*

The studied pharmaceutical formulations were acquired from the drug manufacturer, Yegna Manojavam Drugs and Chemicals Ltd., Nalgonda, India. In particular, the powder samples were as follows: Cetirizine dihydrochloride, Cipro pure, Metformin hydrochloride and Ciprofloxacin hydrochloride, where the active pharmaceutical ingredients (API) in these drug samples are: C_21_H_27_O_3_N_2_Cl_3_; C_17_H_19_ClFN_3_O_3_.H_2_O; C_4_H_12_ClN_5_; and C_17_H_18_FN_3_O_3_.HCl, respectively. The choice of these formulations was governed by the similarity in their chemical composition (especially Cipro pure and Ciprofloxacin hydrochloride) and the ease of local availability. The acquired powder samples were pressed into pellets with a constant pressure of 5 tons for *ca.* 3 minutes using a hydraulic pressure machine. Pelletizing the powder is of significance as it typically causes a dramatic improvement in the reproducibility of the LIBS data [1]. Such an increase can be attributed to the improved rigidity of the pellets in comparison to the powders, which ensures that the position of the focal spot is almost unchanged for all the laser pulses arriving at the pellet surface during a data acquisition cycle. Furthermore, the pellet forms are more representative of the solid dosage forms available commercially and thus the variability encountered in the pellet experiments are expected to closely simulate real-world conditions.

*Instrumentation and Data Acquisition*

The spectroscopic measurements were performed using a home-built LIBS system with an excitation light of 532nm from a solid-state frequency-doubled Nd:YAG module (Spit light 1200, InnoLas LaserGmbH, Germany). The laser was focused on to the sample using an 80 mm convex lens. The emission signal was collected using a separate lens system and coupled to the compact Ocean Optics MAYA2000 unit, which consists of a f/4 symmetric crossed Czerny-Turner spectrometer and a non-gated, back-thinned CCD detector (Hamamatsu S10420). The integration time per LIBS acquisition was set to cover substantially more than the maximum possible lifetime of the plasma (10 μs). A notch filter was used in the collection arm to suppress the interference from the excitation signal at 532 nm. Nevertheless, due to the intensity of the excitation (and the lack of gating in our instrument), a 30 nm wide band surrounding the laser line was excluded to avoid the associated spectral signature from all subsequent analysis.

For spectral acquisition, a 2D motion stage was used to enable investigation of multiple sites of the pellets. Since relatively large variations in composition are often encountered from site to site on a sample (due to the difficulty of blending solid particles exhibiting different size distributions and dissimilar bulk properties), representative analysis of the pellet imposes sampling constraints in terms of minimum number of sites and site-to-site distance. In our experiments, a set of 110 spectra was acquired from multiple sites of each drug pellet. It also bears mention that the laser was focused slightly inside the surface of the pellet to improve spectral reproducibility. It has been previously noted that such shift in focal spot enhances the precision of measurements because a smaller volume of air is excited in this case in relation to when the focal spot is on the sample surface [2,3]. Additionally, the shot to shot fluctuations in emission intensity, arising from the non-uniform distribution of the various constituents in the sample, are less because the spatial region covered by the laser spot in the sample in this case is more than the region covered when focal spot is on the surface of the pellet.

*Multivariate Data Analysis*

The acquired LIBS spectral data was first subjected to pre-processing steps to ensure suitability and consistency for classification purposes. The pre-processing steps included wavelength calibration using standard emission lines, selection of appropriate wavelength region, cosmic ray removal and background removal, as applicable. In each spectrum, 300-1000 nm region was considered for the analysis as they contained the primary features of interest for the samples. As the cosmic spikes on the CCD detector do not exhibit a temporal or site-to-site correlation these were identified by detecting points across multiple frames with significant intensity deviation. Here, significant intensity deviation was considered as 2.22 times the interquartile range (*i.e.* 3σ) above the third quartile for the intensity distribution in the specific spectral channel. Following identification, the cosmic rays were removed by substituting the average value of the neighboring spectral channels without the cosmic ray. In some cases, the final pre-processing step was removal of a best-fit polynomial that mimics the early plasma emission background [4]. However, as detailed in the main manuscript, this was used for investigating the impact of the background on quantitative analysis and was therefore only applied in a select set of cases. Therefore, unless otherwise mentioned, all the LIBS spectra were subjected to algorithm development without removal of background.

Principal component analysis (PCA) was used to reduce the number of dimensions of the spectral data, which in turn enables the visualization of the key features and the clustering behavior of the samples along these new dimensions. PCA utilizes an orthogonal transformation to convert a set of observations of closely correlated variables into a set of values of uncorrelated variables called principal components (PC). Expectedly, the first few principal components accounted for a high degree of the net variance in our dataset.

Prior to development of the classification algorithm, spectral outlier detection and removal were undertaken by using hierarchical clustering, specifically dendrogram analysis. Dendrograms were constructed for each class based on the correlation of distances. In a dendrogram, the objects are organized in a row according to their similarities, where the vertical axis represents the similarity measure at which each successive object joins a group [5]. In our analysis, the correlation values were assessed using a normal distribution and a 2σ cutoff was established for outlier rejection. Approximately 8% of the data points were removed from further data analysis. In particular, the number of spectra retained after dendrogram analysis were: Cetirizine dihydrochloride - 106; Cipro pure - 106; Metformin hydrochloride - 94; Ciprofloxacin hydrochloride - 97. Significantly, dendrogram analysis also provided a method to compare and contrast the clustering pattern observed in PCA.

Finally, three classification methods, namely SIMCA, PLSDA and ANN, were used to develop segmentation algorithms. The first two classifiers were also used in analyzing gated LIBS data from pharmaceutical samples in our laboratory’s previous reports [6, 7]. Concisely, SIMCA is a supervised data classification method that incorporates the application of PCA as a first step (to provide dimensionality reduction as well as suppression of white noise). The central idea of soft modeling is that the classifier can identify samples as belonging to multiple (overlapping) classes and is not constrained to producing a classification of samples into strictly discrete (non-overlapping) classes. Here, each class was independently modeled using PCA, where, the number of principal components for each class in the training samples was determined by leave-one-out cross-validation in order to balance between explaining maximum variance in the dataset and removing the noise-laden PCs. Subsequent to development of the individual PC models, the class distance(s) of the samples from the respective PC models was computed [7]. An average distance metric was calculated for each class (from the associated training samples) to determine a critical distance for classification. Finally, the SIMCA-derived model was used to classify the test samples by comparing the residual variance of these samples with the mean residual variance of the training samples belonging to a specific class. Also, an unclassification criterion was introduced to avoid the misclassification of potential samples that are far from the center of any of the PC models. In particular, it was assumed that the distances of the training data to the center of the corresponding PC model follow a normal distribution. This distribution was then utilized to compute the probability of class membership of each test sample, given the distance of the test spectrum to the center of the different classes. In the event that the membership probability for every class was observed to be less than 0.3% (*i.e.* beyond a 3σ cutoff), it was designated as an "unclassified" sample. For our computations, a modified and extended version of the LIBRA toolbox originally developed by Verboven and Hubert was used [8]. In contrast to SIMCA, PLS-DA involves partial least squares analysis (PLS) analysis for key feature (loading vector) selection and concomitant reduction of noise [7]. The central focus of the PLS-DA algorithm is to obtain optimal separation between each class by fitting one global model to the spectral dataset. This boosts its capability to distinguish between samples even when intra-class variability is comparable to the inter-class variability.

Finally, artificial neural networks (ANN) belong to a class of nonlinear, non-parametric classification and predictive methods, which have lately received attention in LIBS spectral analysis. For example, it has been used to predict chromium element concentration in soils [9], concentration of vanadium and nickel in complex crude oils [10] and for material identification in plant materials [11]. Here, a two-layer feed-forward network model was used for classification consisting of one input layer, one hidden layer and one output layer. The input layer comprises of raw data intensity values at various wavelengths. Sigmoid functions were used as neurons for the input and hidden layers. The final output consists of binary encoding used as target, where 1 is for correct assignment and 0 for absent. The ANN was trained with a classical scaled conjugate gradient back-propagation algorithm. The data division control was in-built in the toolbox. Out of the total spectral data, 70% were presented to the network during training, and the network was adjusted according to its error. 15% of the data was used to measure network generalization and to halt training indicating an increase in the mean square error of the validation samples. The remaining 15% were held out of the training and validation process and therefore provide an independent measure of network performance.

**References:**

1. Lal B, Zheng H, Yueh F-Y, Singh JP (2004) Parametric Study of Pellets for Elemental Analysis with Laser-Induced Breakdown Spectroscopy. Appl Opt 43: 2792-2797.

2. Krasniker R, Bulatov V, Schechter I (2001) Study of matrix effects in laser plasma spectroscopy by shock wave propagation. Spectrochimica Acta Part B: Atomic Spectroscopy 56: 609-618.

3. Bassiotis I, Diamantopoulou A, Giannoudakos A, Roubani-Kalantzopoulou F, Kompitsas M (2001) Effects of experimental parameters in quantitative analysis of steel alloy by laser-induced breakdown spectroscopy. Spectrochimica Acta Part B: Atomic Spectroscopy 56: 671-683.

4. Mueller M, Gornushkin IB, Florek S, Mory D, Panne U (2007) Approach to Detection in Laser-Induced Breakdown Spectroscopy. Analytical Chemistry 79: 4419-4426.

5. Brereton RG (2003) Pattern Recognition. Chemometrics: John Wiley & Sons, Ltd. pp. 183-269.

6. Myakalwar AK, Sreedhar S, Barman I, Dingari NC, Venugopal Rao S, et al. (2011) Laser-induced breakdown spectroscopy-based investigation and classification of pharmaceutical tablets using multivariate chemometric analysis. Talanta 87: 53-59.

7. Dingari NC, Barman I, Myakalwar AK, Tewari SP, Kumar Gundawar M (2012) Incorporation of Support Vector Machines in the LIBS Toolbox for Sensitive and Robust Classification Amidst Unexpected Sample and System Variability. Analytical Chemistry 84: 2686-2694.

8. Verboven S, Hubert M (2005) LIBRA: a MATLAB library for robust analysis. Chemometrics and Intelligent Laboratory Systems 75: 127-136.

9. Sirven J-B, Bousquet B, Canioni L, Sarger L, Tellier S, et al. (2006) Qualitative and quantitative investigation of chromium-polluted soils by laser-induced breakdown spectroscopy combined with neural networks analysis. Analytical and bioanalytical chemistry 385: 256-262.

10. Tarazona JL, Guerrero J, Cabanzo R, Mejía-Ospino E (2012) Construction of a predictive model for concentration of nickel and vanadium in vacuum residues of crude oils using artificial neural networks and LIBS. Applied Optics 51: B108-B114.

11. Nunes LC, da Silva GA, Trevizan LC, Santos Júnior D, Poppi RJ, et al. (2009) Simultaneous optimization by neuro-genetic approach for analysis of plant materials by laser induced breakdown spectroscopy. Spectrochimica Acta Part B: Atomic Spectroscopy 64: 565-572.
